# Supplementary material for: Increasing the spatial bandwidth product in light field microscopy with remote scanning
Source: Biomed Opt Express. 2025 Feb 13;16(3):1062–70. doi: 10.1364/BOE.544498 (PMC11919350; doi:10.1364/BOE.544498)
Supplement: Supplementary file 1 [file boe-16-3-1062-s001.pdf]

Supplemental Document

Biomedical Optics EXPRESS

Increasing the spatial bandwidth product in light field microscopy with remote scanning: supplement

AYMERICK BAZIN[1,2] AND AMAURY BADON[1,2,*]

[1] *LP2N, Laboratoire Photonique Numéerique et Nanosciences, Univ. Bordeaux, F-33400 Talence, France*
[2] *Institut d'Optique Graduate School & CNRS UMR 5298, F-33400 Talence, France*
[*] *amaury.badon@cnrs.fr*

This supplement published with Optica Publishing Group on 13 February 2025 by The Authors under the terms of the Creative Commons Attribution 4.0 License in the format provided by the authors and unedited. Further distribution of this work must maintain attribution to the author(s) and the published article's title, journal citation, and DOI.

Supplement DOI: https://doi.org/10.6084/m9.figshare.28270721

Parent Article DOI: https://doi.org/10.1364/BOE.544498

Increasing the spatial bandwidth product in Light Field Microscopy with remote scanning: supplemental document

This document provides supplementary information to "Increasing the spatial bandwidth product in Light Field Microscopy with remote scanning," . It provides details related to the experimental setup, image pre-processing, volume reconstruction, resolution enhancement and field of view increase.

1. EXPERIMENTAL SETUP

The light-field imaging system as illustrated in Figure S1 is built upon a commercially available inverted microscope (Zeiss, Axiovert 200M) configured with a 20x/0.75-NA air objective (Nikon, 20X CFI Super Fluor). The setup is equipped with a with light halogen lamp (HAL 100, Zeiss) for bright field illumination and an 4-color light source for epifluorescence illumination (pE-2, CoolLed, wavelength 400, 490, 550 and 635 nm, 100 mW output power). The setup is equipped with a scientific complementary metal-oxide-semiconductor (sCMOS) camera (Hammamatsu, Orca Flash 4.0) which has 2048 × 2048 pixels with a 6.5-µm pixel pitch size and a 100-Hz speed in full frame. A relay lens system, comprising L_1 with a focal length of 100 mm and L_2 with a focal length of 200 mm, is used to match the numerical aperture (NA) of the objective with the NA of the microlens array (Viavi, MLA-S100-f21). This condition is expressed by the relationship $\frac{1}{2\times f^{\#}} = \frac{NA_{obj}}{M}$, where M is the magnification between the focal plane and the MLA (microscope and relay lens system) and $f^{\#}$ is the f-number of the MLA. The stirring mirror (Optotune, MR-E2) is placed in the focal plane of L_1 which corresponds to the Fourier plane of the system. A second relay system (L_3, f=180 mm and L_4, f=165 mm, magnification = 0.92) conjugates the back focal plane of the MLA with the camera sensor. In this configuration, each microlens is mapped onto approximately 14 × 14 camera pixels which sample the angular information. Regarding the spatial information, the lateral resolution of the system is defined by twice the physical pitch of a microlens divided by the magnification M of the optical system. Here, we obtain 3.1 µm.

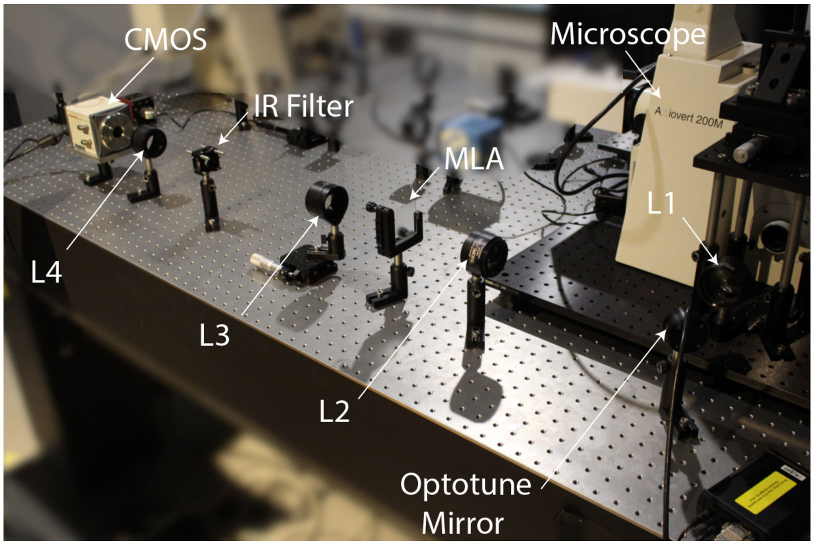

Fig. S1. Photo of the experimental setup. Here we see the light field module which is placed at the output of a commercial microscope body.

2. IMAGE PRE-PROCESSING

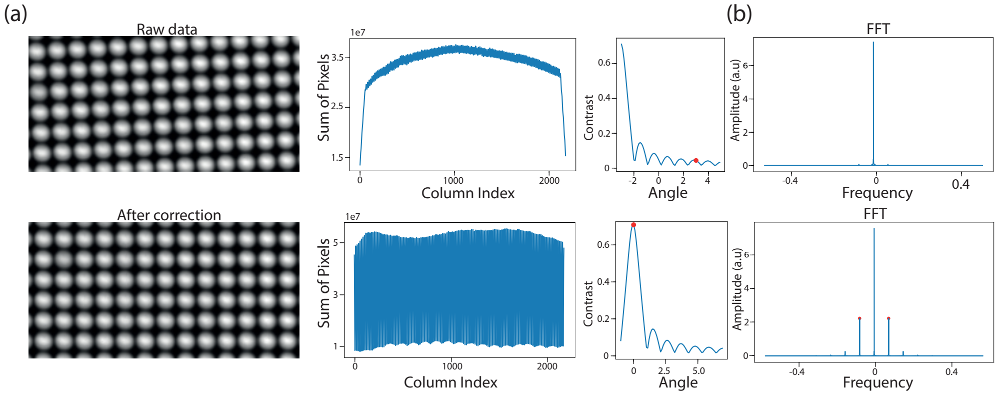

Fig. S2. Automatic angle correction and microlens pitch detection. (a) A raw light field image is shown with both uncorrected and corrected rotation angles, the plots illustrate the relationship between angle and contrast of the intensity profile, demonstrating that contrast is maximized at 0° and decreases with a 3° correction. (b) The lens pitch is detected in the frequency domain.

A calibration of the raw light field images must be performed to associate the raw data with a 4D array $[x, y, u, v]$, where x and y denote the index of microlenses in the horizontal and vertical directions respectively and u and v indicate the angular positions in the horizontal and vertical directions respectively. The aim of this calibration step is (i) to correct for possible rotation of the MLA, (ii) to find the pitch in the image and (iii) to detect the centers of each microlens.

Due to mounting imperfections of the MLA, a rotated image can be obtained onto the camera. A correction step is necessary to obtain correct horizontal and vertical axes. The raw light field image corresponds to the characteristic grid structure of the MLA, alternating between bright and dark spots as described in [1]. The sum of the pixel intensity in the raw image along each column produces a 1D signal representing an intensity profile. The contrast of this intensity profile varies with the rotation of the image and reaches its maximum when the image is perfectly aligned. Here, contrast is defined as the ratio of amplitude (the difference between the maximum and minimum intensity) to the sum of the maximum and minimum intensities. To find the optimal rotation angle , the image is rotated in increments of $0.1°$ within the range of $[-5°, 5°]$, which is progressively narrowed. Optimal correction factor is obtained for the highest contrast value, as shown in Figure S2 (a). To save computation time, the contrast can be calculated on a cropped section of the image.

Once the raw image is corrected for a possible rotation, the pitch of the MLA in the camera plane can be estimated. The intensity profile of the corrected image is supposed to be periodic and to match with the pitch between microlenses. By computing the Fast Fourier Transform (FFT) of this signal and pinpointing the highest frequency peak (excluding 0, which corresponds to the signal's mean value), we can estimate the pitch between two microlenses, as shown in Figure S2 (b). The reciprocal of this period is equal to the microlens pitch in the spatial domain. The image is then resized to achieve an integer and odd pitch value, which facilitate 3D image reconstruction.

Finally, detecting the microlens centers is the final step in accurately parameterizing the 4D array of the raw image. This is important to correctly assign angles for each spatial location. Without this step, artifacts appears in volumetric reconstruction, especially for large depths. For this step, the image is binarized, and we define two empty arrays to store the coordinates of the microlens centers in x and y. We start with initial coordinates x_0 and y_0, which approximately correspond to the first microlens in the top-left corner of the raw image, serving as the starting point. We extract a 15×15 pixels sub-image centered around the estimated microlens centers and we apply a center of mass function from SciPy that computes the centroid of this cropped binarized image [2]. The correct position of the center is obtained. We then iteratively add 15

pixels to move to the next center, first in X till the end of the row and then in Y on the raw image. The centered sub-images are stored in a 4D array that contains all the 15×15 pixels sub-images. Figure S3 (b) illustrates the process on a reference image without a sample, which is used to create the microlens center maps. These coordinates are then applied to a sample image taken in bright-field or fluorescence.

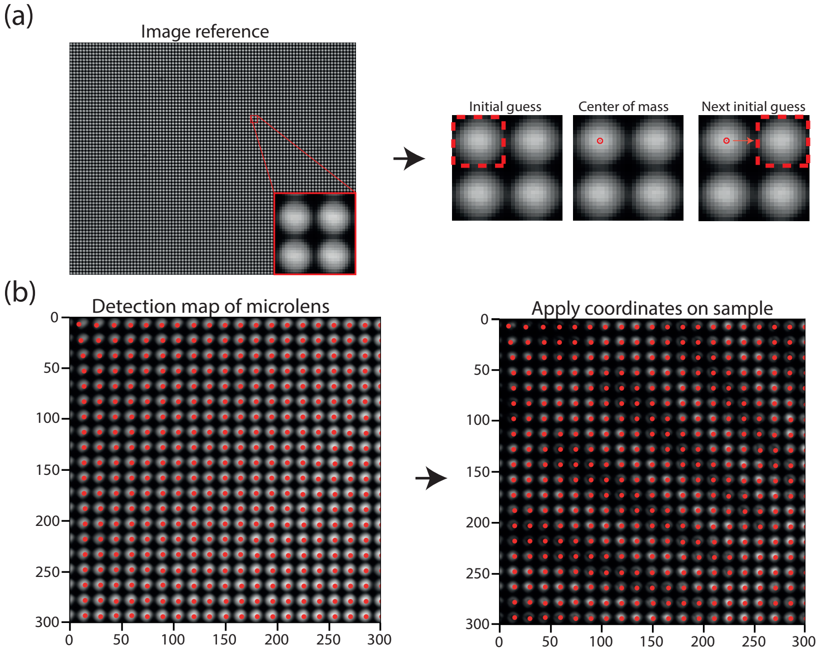

Fig. S3. Calibration of microlens centering. (a) The center of the microlenses is detected using the local center of mass of a microlens and reiterated over the entire reference image. (b) The coordinates of the microlens centers in the reference image are applied to an image with sample.

Optionally, a normalization of the raw image can be performed to enhance the contrast. The difference between the sample images and the reference image is computed. This difference is then divided by the reference image, to which a small constant, ϵ, has been added to prevent division by zero.

3. VOLUME RECONSTRUCTION AND 3D DECONVOLUTION

Once pre-processing is complete, we obtain a 4D array, LFM[x, y, u, v], a structure well adapted to extract positions and perspectives that will be used to create volumetric images.

Perspectives are extracted from the 4D table by selecting all microlens pixels indexed by (u, v) in LFM[:, :, u, v] as shown in Figure S4 (a). The perspectives are then processed by a shift and sum algorithm [3]. Before summing the different perspectives, the concept is to apply an appropriate shift in X and Y to each perspectives by an amount that depends on the depth and the index of the perspectives (u, v). We define a coefficient C that depends on the depth and a 2D map which depends on the perspectives indexes. Figure S4 (b) displays the absolute value of this 2D map multiplied by the coefficient C for three different depths. While the 2D map is purely geometrical, the coefficient C is determined experimentally. To do so, a sample is physically defocused at a known depth. Using the central perspective as a reference, the shift between a given perspective is estimated. This shift is measured at various depths, providing the evolution of this shift as a function of depth for a given perspective. The slope of this shift with respect to depth gives the shift factor C.

Figure S4 (c) illustrates the process for different values of C to generate a stack of 2D images at various depths, which together form a volumetric image.

Finally, 3D deconvolution can be applied onto the data to restore the resolution and signal

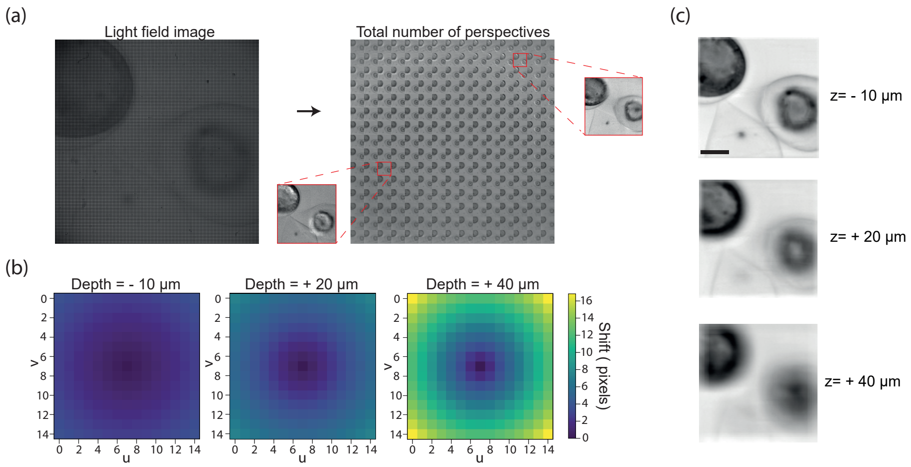

Fig. S4. Shift and Sum algorithm principle. (a) From the calibrated LF image, we extract the 15 × 15 perspectives. (b) Shift of perspectives along X and Y as a function of depth for -10 microns, 20 microns, and 40 microns. (c) Sum of shifted perspectives to obtain a digitally refocused image at different depths.

far from the focus. We define the experimental point spread function (PSF) as a cropped region corresponding to a sub-resolution sample. We then process the 3D data with a 3D deconvolution plugin in ImageJ [4] with the experimental PSF and 2 iterations.

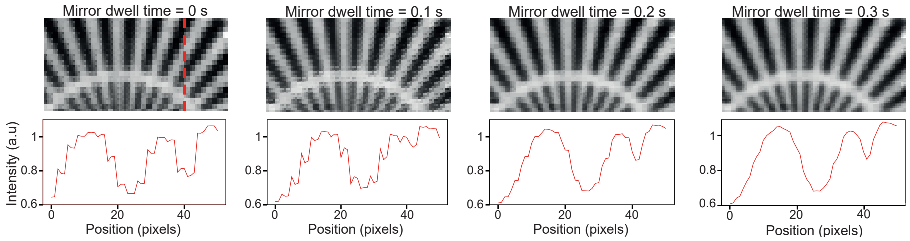

Fig. S5. Intensity profile for different mirror dwell times. As the dwell time increases, the profile becomes smoother, indicating better resolution with a more even distribution of pixels.

4. RESOLUTION ENHANCEMENT

The native lateral resolution in LFM is limited by the size of the microlenses divided by the magnification M of the optical system. Here, we detail our technique to overcome this limitation.

First, images are captured by shifting the detected region with a tilting mirror placed in the Fourier plane, where a tilting results in a shift in the image plane. Nine images are captured, each shifted by a third of a microlens in x and y, corresponding to a shift of 5 pixels on the camera sensor. Mirror control is performed with the Optotune library in Python. A 3x3 grid defines the mirror displacement pattern for acquiring these 9 images. Calibration and 3D reconstruction are performed individually on each image, as detailed in the previous sections. The 9 volumetric images are organized as stack of 2D images, each image initially containing 140 x 140 pixels. According to the acquisition pattern, each pixel from the conventional images is placed into a 420 x 420 matrix, creating a refocused image at a higher resolution. This process is repeated for all depths to generate a 3D image composed of 30 slices, each with dimensions of 420 x 420 pixels.

The mirror's settling time and the shift step between two images are crucial parameters for improving resolution. The settling time was determined by testing various durations ranging from 0 to 0.5 seconds, increasing in increments of 0.1 seconds. An intensity profile was plotted along the Y-axis for each settling time and compared. Figure S5 displays the "jagged" effect observed with settling times below 0.3 seconds, where the mirror doesn't have sufficient time to properly position itself between image captures. This results in reduced efficiency in pixel

extraction and placement, leading to less effective microlens sampling and a poor image resolution of 420 x 420 pixels.

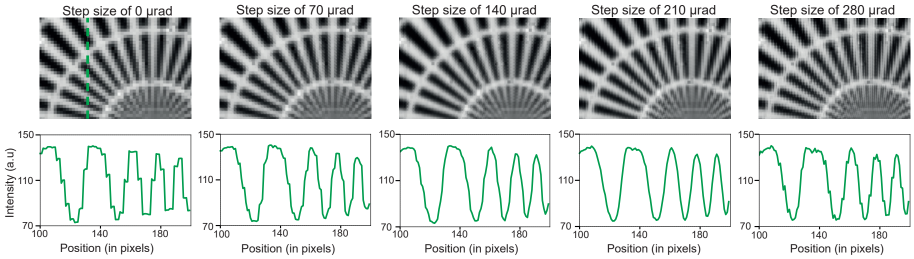

Fig. S6. The intensity profiles for different mirror steps ranging from 0 to 280 microradians, with increments of 70 microradians, show decreasing smoothness as the steps deviate from 140 microradians. This value represents the optimal step size for sampling half a microlens (5 pixels) on the camera.

The shift step between two images in both X and Y directions should correspond to 5 pixels on the camera, which is equivalent to a few microradians for the mirror. This step is defined by increments ranging from 0 µrad to 280 µrad, increasing by 70 µrad increments, as illustrated in Figure S6. The optimal step is selected based on the smoothest intensity profile, visually corresponding to the image with the least aliasing and the best sampling of the microlenses during imaging with the mirror.

Next, once the shift between two images is correctly chosen, we tried to increase the number of laterally shifted low resolution images to further improve the lateral resolution. Theoretically, lateral resolution can be improved down to the diffraction limit, here 410 nm, by using a 8 x 8 scanning scheme. In addition to the important acquisition time, this also requires an excellent accuracy for the tilting mirror position. As illustrated on figure S7, no significant resolution improvement is observed for more than 3 images in each direction.

Finally, we implemented a more robust and sophisticated method to obtain a higher resolution image G_f from multiple low-resolution images I_k. As proposed in this work [5], we defined a motion operator F, a downsampling opertor D and a high-resolution can be obtained using the following minimization scheme :

$$G_f = \underset{G}{\text{ArgMin}} \left[\sum_{k=1}^{N} \|DF_k G - I_k\| \right] \tag{S1}$$

As displayed in figure S8, no significant improvement is visible between the simple linear combination of low-resolution image and the one obtained using the minimization approach. As the latter method is relatively long (few minutes per image), we decided to use only simplest and fastest solution.

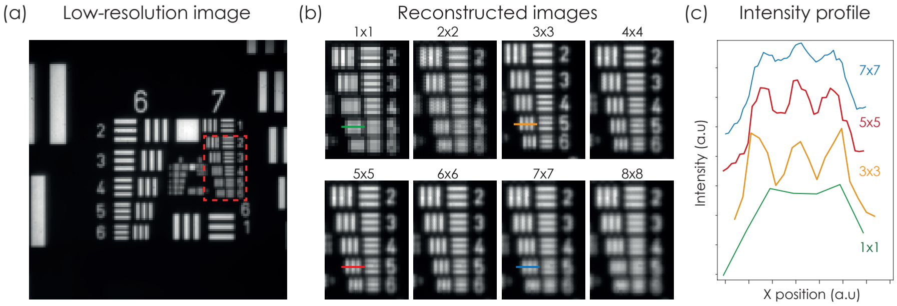

Fig. S7. Resolution enhancement with multi-frame acquisition. (a) Low resolution image of an USAF 1951 resolution target in bright-field mode. (b) Cropped images corresponding to the region in red in (a) for various multi-frame acquisition, ranging from 1 x 1 to 8 x 8. (c) Comparison of intensity profiles corresponding to the group 7 element 5 of the USAF resolution target.

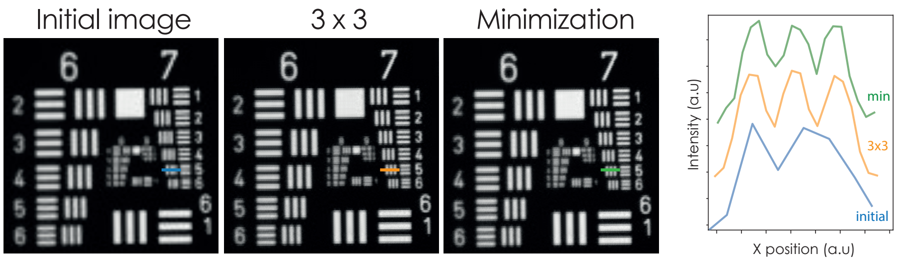

Fig. S8. Comparison of the reconstruction process performances. Left, initial low-resolution image. Center, our reconstruction using the simple pixel manipulation. Right, image obtained using the minimization approach.

Despite these clear evidences that 3x3 scanning improves the resolution of the LFM, we captured images of 1 µm fluorescent beads dispersed in 3D in agarose. From a single acquisition, both in 1X1 and 3X3 mode, we reconstructed 3D volumes to estimate the 3D PSF of our system. These results are displayed in figures S9 andS10.

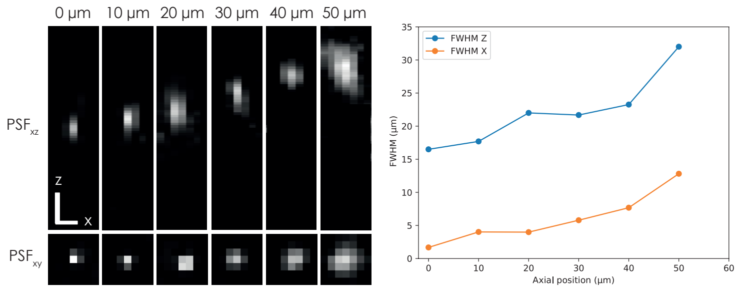

Fig. S9. Left, 3D PSF measured with 1 µm fluorescent beads. Scale bar in X, 10 µm. Scale bar along Z, 25 µm. Right, full width at half maximum obtained from the PSF in X and Z directions.

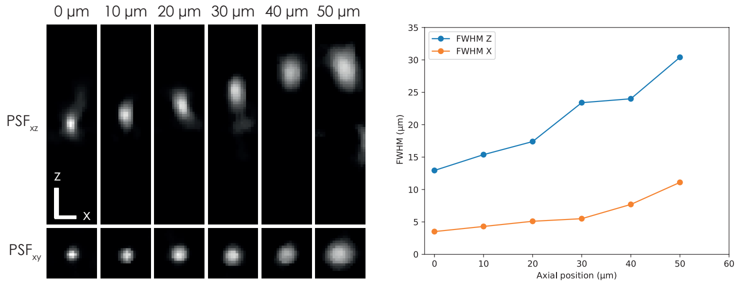

Fig. S10. Left, 3D PSF measured with 1 µm fluorescent beads in the 3x3 scanning mode. Scale bar in X, 10 µm. Scale bar along Z, 25 µm. Right, full width at half maximum obtained from the PSF in X and Z directions.

Surprisingly, no improvement of the FWHM is observed when we directly compare the results in 1X1 and 3X3 mode, both laterally and axially (see figure S11).

This is not in line with the results obtained with the USAF resolution target. Thus, we then performed imaging experiments to distinguish closed sub-diffraction beads. As seen on figure S12, beads that are too closed to be distinguished according to Rayleigh criterion in the 1X1 scanning mode become clearly resolved in the 3X3, demonstrating the improvement in lateral resolution. Here, beads separated by a distance of around 3 µm are distinguishable in the 3X3 scanning mode.

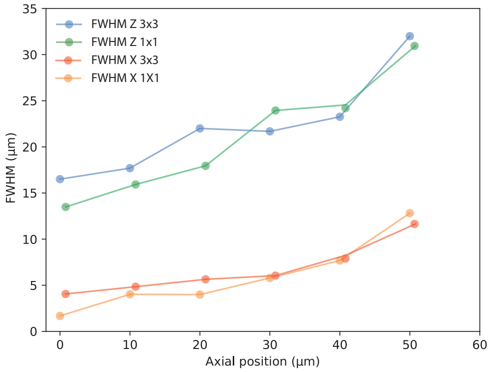

Fig. S11. Comparison of the FWHM obtained in the lateral and axial directions for both 1X1 and 3X3 scanning mode.

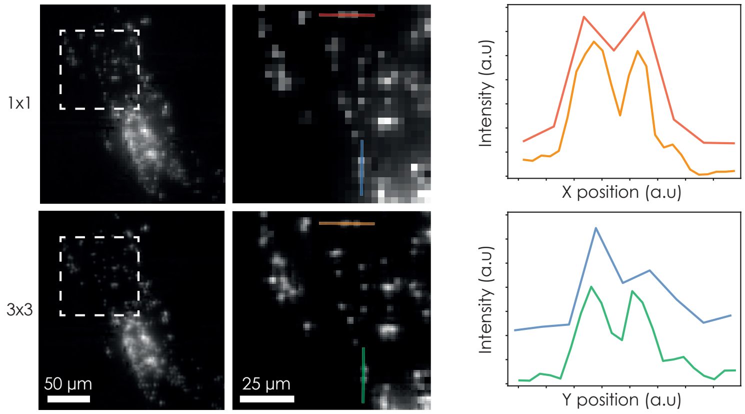

Fig. S12. Imaging of closely spaced sub-diffraction beads. Left, field of view captured in LFM. Center, close up view of the region highlighted with the white dotted lines. Right, intensity profiles corresponding to the colored continuous line in the close up views.

5. FIELD OF VIEW INCREASE

In LFM, the FOV in limited by the number of camera pixels. Here, we provide details on our technique to enlarge the FOV without moving the sample by using a motorized tilting mirror in the Fourier plane upstream the MLA.

First, the calibration of the stitching procedure is performed using a USAF 1951 resolution target sample in bright field mode. A sequence of 25 images, arranged as 5 along the X-axis and 5 along the Y-axis, is captured to cover the FOV of the objective (see Figure S13 (a)). The images are captured by shifting the mirror using coordinates with the Optotune library in Python, with a 0.1 second pause between each capture. The overlap between the images, estimated to be around 30% to ensure sufficient overlap for image stitching, is experimentally determined by assessing the diameter of the camera's FOV and calculating the number of images that are needed to cover the entire objective field of view. Each image is individually processed through calibration and 3D reconstruction to obtain 25 stacks of digitally refocused 2D images.

After this acquisition, the stitching is divided into two steps. Images are first assembled 5 by 5 along the X-axis using the Fiji stitching plugin [6] in Python through PyimageJ [7]. The images are merged using a linear blend, gradually adjusting the intensity between images. The coordinates are calculated and stored in 5 text files corresponding to the 5 image assemblies. These files can be used for other samples without recalculating the coordinates, thereby saving a significant amount of computation time.

The 5 images stitched along the X-axis are then individually rotated at different angles to facilitate the stitching of images along the Y-axis. This rotation might appear for large angles of

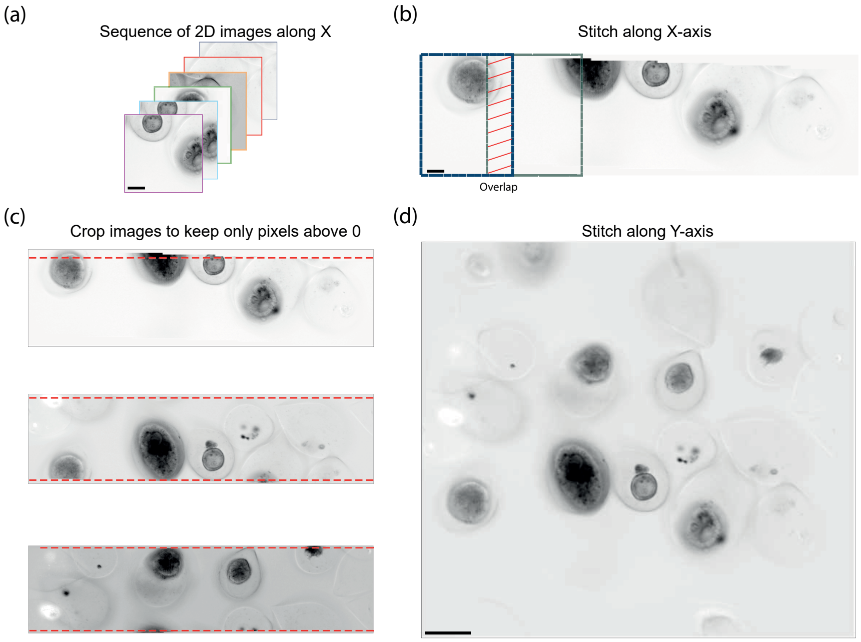

Fig. S13. Stitching principle. (a) Twenty-five images are captured by the camera, each covering a different region of the field of view of the objective. (b) The images overlap by 30%, which the plugin uses to stitch them together along the X-axis. (c) To improve the quality of the stitching along the Y-axis, the images are cropped to remove any null pixels at the top and bottom. (d) These cropped images, containing only positive pixels, are then stitched along the Y-axis with a 20% overlap.

the mirror, when a tilt in the Fourier plane is not perfectly converted into a shift in the camera plane. Additionally, since the images are assembled into a mosaic, null pixels may appear at the top and bottom of the image. These null pixels hinder with the stitching process along the Y-axis because the plugin cannot perform image blending by comparing intensities; therefore, the null pixels common to the images are stitched randomly. To overcome this, we crop the images by automatically detecting and removing the null pixels at the top and bottom, retaining only the non-null information as illustrated in Figure S13 (b). Automatic cropping is applied once to the five images assembled according to X-axis. The number of pixels removed from the top and bottom is recorded and then applied to subsequent samples.

Finally, a final stitching process is performed to assemble the images along the Y-axis, with a 20% overlap between each image. This method produces a final image that covers the entire field of view of the objective. To ensure seamless integration, linear blending is applied at the overlapping sections. The coordinates used for this final assembly are saved in a sixth text file. These coordinates can be reused for other samples, allowing for significant computation time savings.

It is legitimate to ask whether enlarging the field of view might not result in a deterioration in the performance of the experimental set-up. First, we imaged in bright field mode, a USAF resolution target placed at various positions in the microscope objective FOV. As seen on figure S14, we did not observe degradation of the lateral resolution even for positions outside of the FOV defined by the field number of the objective (here FN=22, so the theoretical FOV is equal to 1.1 mm), mainly because we are far from the diffraction limit.

Secondly, we imaged in epifluorescence mode 1 µm beads for various positions inside the microscope FOV. Due to the limited extent of the illumination lamp, we are limited to a diameter of 1.2 mm. As seen on figure S15, the lateral and axial resolution are almost constant over the entire accessible FOV, demonstrating that remote scanning does not degrade the optical performances of our apparatus.

All the codes used in this project are available here [8].

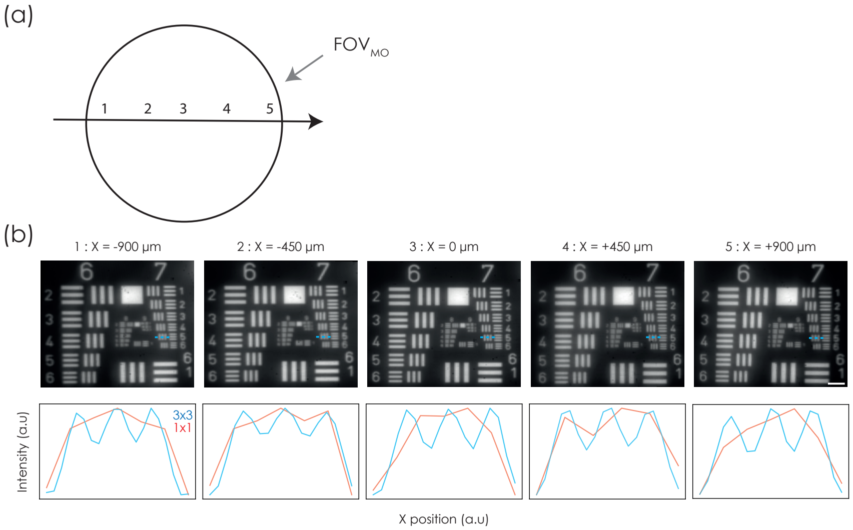

Fig. S14. Estimation of the lateral resolution in bright field mode for various positions across the field of view. (a) Positions of the USAF resolution target across the microscope objective field of view. (b) Bright field images and intensity profiles corresponding to the group 7 element 5 for both 1X1 and 3X3 scanning mode.

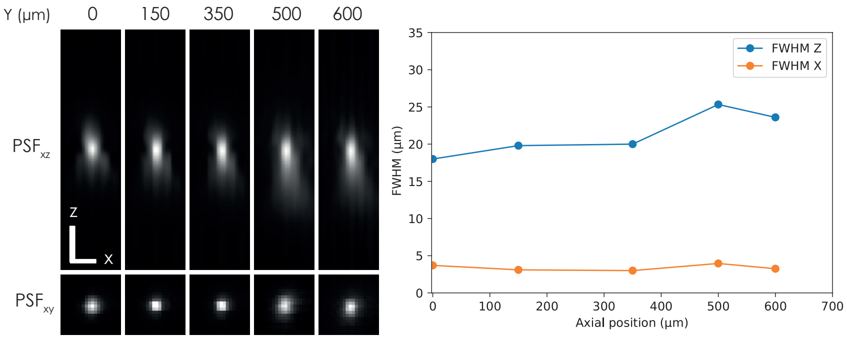

Fig. S15. Left, point spread function measurement with 1 µm fluorescent beads for various positions across the field of view. Right, full width at half maximum obtained from the PSF in X and Z directions.

REFERENCES

1. P. Song, H. V. Jadan, C. L. Howe, *et al.*, "3d localization for light-field microscopy via convolutional sparse coding on epipolar images," IEEE Transactions on Comput. Imaging **6**, 1017–1032 (2020).
2. P. Virtanen, R. Gommers, T. E. Oliphant, *et al.*, "Scipy 1.0: fundamental algorithms for scientific computing in python," Nat. methods **17**, 261–272 (2020).
3. M. Levoy, R. Ng, A. Adams, *et al.*, "Light field microscopy," in *ACM SIGGRAPH 2006 Papers on - SIGGRAPH '06*, (ACM Press, 2006), SIGGRAPH '06.
4. E. Crowell and D. J. White, "Deconvolution," https://imagej.net/imaging/deconvolution.
5. K. Shen, H. Lu, S. Baig, and M. R. Wang, "Improving lateral resolution and image quality of optical coherence tomography by the multi-frame superresolution technique for 3d tissue imaging," Biomed. optics express **8**, 4887–4918 (2017).
6. S. Preibisch, S. Saalfeld, and P. Tomancak, "Globally optimal stitching of tiled 3d microscopic image acquisitions," Bioinformatics **25**, 1463–1465 (2009).
7. C. T. Rueden, M. C. Hiner, E. L. Evans III, *et al.*, "Pyimagej: A library for integrating imagej and python," Nat. methods **19**, 1326–1327 (2022).

8. https://github.com/BiOflab/Light_field_microscopy.
